# Elevation of sulfatides in ovarian cancer: An integrated transcriptomic and lipidomic analysis including tissue-imaging mass spectrometry

**DOI:** 10.1186/1476-4598-9-186

**Published:** 2010-07-12

**Authors:** Ying Liu, Yanfeng Chen, Amin Momin, Rebecca Shaner, Elaine Wang, Nathan J Bowen, Lilya V Matyunina, L DeEtte Walker, John F McDonald, M Cameron Sullards, Alfred H Merrill

**Affiliations:** 1 School of Biology and the Petit Institute for Bioscience and Bioengineering, Georgia Institute of Technology, 315 Ferst Drive, Atlanta, Georgia 30332-0363, USA; 2 School of Chemistry and Biochemistry and the Petit Institute for Bioscience and Bioengineering, Georgia Institute of Technology, 315 Ferst Drive, Atlanta, Georgia 30332-0363, USA

## Abstract

**Background:**

Sulfatides (ST) are a category of sulfated galactosylceramides (GalCer) that are elevated in many types of cancer including, possibly, ovarian cancer. Previous evidence for elevation of ST in ovarian cancer was based on a colorimetric reagent that does not provide structural details and can also react with other lipids. Therefore, this study utilized mass spectrometry for a structure-specific and quantitative analysis of the types, amounts, and tissue localization of ST in ovarian cancer, and combined these findings with analysis of mRNAs for the relevant enzymes of ST metabolism to explore possible mechanisms.

**Results:**

Analysis of 12 ovarian tissues graded as histologically normal or having epithelial ovarian tumors by liquid chromatography electrospray ionization-tandem mass spectrometry (LC ESI-MS/MS) established that most tumor-bearing tissues have higher amounts of ST. Because ovarian cancer tissues are comprised of many different cell types, histological tissue slices were analyzed by matrix-assisted laser desorption ionization-tissue-imaging MS (MALDI-TIMS). The regions where ST were detected by MALDI-TIMS overlapped with the ovarian epithelial carcinoma as identified by H & E staining and histological scoring. Furthermore, the structures for the most prevalent species observed via MALDI-TIMS (d18:1/C16:0-, d18:1/C24:1- and d18:1/C24:0-ST) were confirmed by MALDI-TIMS/MS, whereas, a neighboring ion(*m/z *885.6) that was not tumor specific was identified as a phosphatidylinositol. Microarray analysis of mRNAs collected using laser capture microdissection revealed that expression of *GalCer synthase *and *Gal3ST1 *(3'-phosphoadenosine-5'-phosphosulfate:GalCer sulfotransferase) were approximately 11- and 3.5-fold higher, respectively, in the ovarian epithelial carcinoma cells versus normal ovarian stromal tissue, and they were 5- and 2.3-fold higher in comparison with normal surface ovarian epithelial cells, which is a likely explanation for the higher ST.

**Conclusions:**

This study combined transcriptomic and lipidomic approaches to establish that sulfatides are elevated in ovarian cancer and should be evaluated further as factors that might be important in ovarian cancer biology and, possibly, as biomarkers.

## Background

Epithelial ovarian cancer is the fourth leading cause of death for women in the United States and has the highest death rate of all gynecological cancer [1]. The 5-year survival rate is less than 30% [2], in part because accurate diagnosis is often not made until it has progressed into more advanced stages. Therefore, knowledge about the molecular changes in ovarian cancer cells might aid both the understanding of the malignant carcinoma progression and the development of strategies for early detection and treatment.

Glycosphingolipids have long been known to be abnormal in many types of cancer [3,4]. One of the categories of glycosphingolipids, sulfatides (ST), has been correlated with poor prognosis in colorectal [5] carcinoma, and found in numerous other types of cancer, including hepatocellular [6], renal [7], and small-cell lung cancers [8]. ST have been suggested to increase in ovarian cancer and possibly to be an early predictor of the disease [9], however, the evidence for elevation of ST in ovarian cancer [9] was based on a colorimetric assay that might have cross-reacted with other lipids (such as cardiolipin, phosphatidylserine or phosphatidylinositol) [10].

This manuscript describes studies that were initiated when a recent analysis of gene expression in epithelial ovarian cancer cells that had been collected using laser capture microdissection [11] provided a data set that included most of the genes for the early steps of sphingolipid metabolism. Examination of the microarray data using a sphingolipid pathway map indicated that epithelial ovarian cancer cells might have elevated ST, which was confirmed by liquid chromatography, electrospray-ionization tandem mass spectrometry (LC ESI-MS/MS) [12] with localization of the ST to the cancer cells using matrix-assisted laser desorption/ionization tissue imaging mass spectrometry (MALDI TIMS) [13].

## Results

### Differences in expression of genes for sphingolipid metabolism between human serous papillary ovarian carcinoma tissue versus normal ovarian stromal tissue

**Figure 1:**
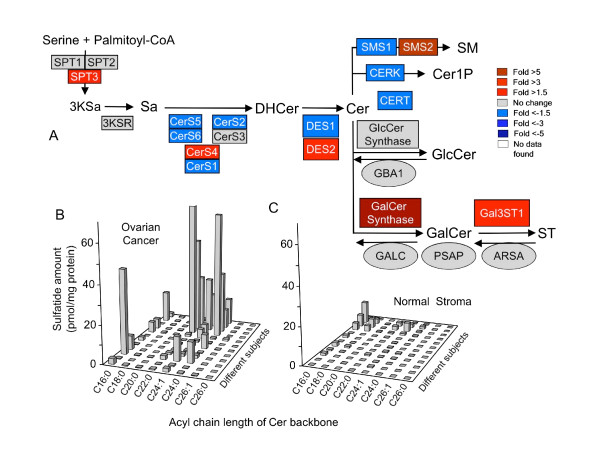
**Differences in the level of expression of genes for sphingolipid biosynthesis through sulfatides, ST, and the amounts of ST in epithelial ovarian tumors versus normal ovarian stromal tissues**. (A) The relative levels of mRNA for the enzymes of the shown steps of the *de novo *sphingolipid biosynthesis pathway were imported into a KEGG style pathway heatmap [15] with fold differences in gene expression for ovarian cancer/normal stromal cells are represented by the color scale (red = higher; blue = lower). In this depiction, the pathway begins in the upper left corner with the condensation of serine and palmitoyl-CoA by serine palmitoyltransferase (SPT, shown for its three known genes) to form 3-ketosphinganine (3KSa) which is converted to sphinganine (Sa) by 3KSa reductase. Sa is N-acylated to dihydroceramide (DHCer) by a family of Cer synthases (CerS), desaturated (by DHCer desaturases, DES), and converted to sphingomyelin (SM) by SM synthases (SMS), phosphorylated to Cer 1-phosphate by Cer kinase (CERK) (this branch of sphingolipid biosynthesis is also thought to involve a Cer transport protein, CERT), or glycosylated to glucosylceramide (GlcCer) or galactosylceramide (GalCer), which can undergo sulfation to ST by GalCer sulfotransferase (Gal3ST1). Also shown are the steps of ST turnover, via aryl sulfatase (ARSA) and galactocerebrosidase (GALC) which operate in concert with prosaposins (PSAP), and GlcCer turnover via glucosylcerebrosidase (GBA1). The heat maps depict the normalized level of expression of these genes in the epithelial ovarian tumors divided by the level of expression on normal stromal tissues averaged for the 12 patients and 8 controls. (B and C) Amounts of the various N-acyl-chain length subspecies of ST as measured by LC ESI-MS/MS for clinically similar patients with ovarian carcinoma (B, n = 12) or normal ovary (C, n = 12).

Using data from a recent study of gene expression in ovarian cancer [11], the fold differences in the mRNAs for enzymes of the early steps of sphingolipid biosynthesis and turnover were calculated and displayed in a heat map format in Figure 1A with the metabolic steps depicted as a KEGG pathway-style diagram [14] that has been recently updated [15]. It is evident from this depiction that the mRNAs for several of the enzymes of ST biosynthesis--most notably GalCer synthase (also called ceramide galactosyltransferase, *UGT8*) and GalCer sulfotransferase (*Gal3ST1*)--are higher for the ovarian carcinoma cells versus normal stromal tissue, whereas those for ST turnover are not different (arylsulfatase, *ARSA*, galactosylceramidase, *GALC*, and possibly the related saposins, based on the pro-saposin mRNA *PSAP*). In addition, mRNAs for two of the three enzymes that also utilize Cer for biosynthesis of other sphingolipids (sphingomyelin synthase 1, *SMS1*, and ceramide kinase, *CERK*) and the Cer transport protein *CERT *appear to be lower for the carcinoma cells versus normal stromal tissue. Therefore, these findings would predict that ovarian carcinoma cells could have higher ST versus normal stromal tissue, as had been suggested by an earlier analysis of ST in this cancer [9].

We were unable to measure ST in the cells isolated by laser capture microdissection because the film used to recover the cells contributed interfering ions, however, it was evident from analysis of tissue samples from 12 patients with the same ovarian cancer diagnosis (Figure 1B) and 12 controls (Figure 1C) that ST are higher in ovarian cancer. The mean ± SE for the total ST (i.e., the sum of the chain lengths) for these groups were 39.8 ± 13.9 pmol/mg tissue for the ovarian cancer patients versus 5.2 ± 2.1 pmol/mg for the controls, which was statistically significant (*P *= 0.006, n = 12 for each by one-tailed Wilcoxon rank sum test).

Two other differences worth noting in Figure 1A are a higher expression of the ceramide syntase 4 (*CerS4*) (2.1-fold) and decreases in all of the other *CerSs *except *CerS3*. Since all of these enzymes act on the same sphingoid base substrate, these levels of expression would be predicted to be functionally equivalent to increases in both *CerS4 *and *CerS3*, which produce Cer with very-long-chain fatty acids (i.e., ≥20 carbon atoms) [16]. In agreement with this prediction, the ST in most of the ovarian epithelial carcinoma tissues (Figure 1B) have predominantly very-long-chain Cer whereas most of the Cer of ST in the normal tissue (Figure 1C) have the C16-backbone. In the cases where the ovarian cancer cells have higher proportions of C16:0-ST, this might reflect individual variation in the expression of the CerS isoforms or perhaps other metabolic processes that impact Cer composition.

The mRNAs for two additional genes are elevated in the ovarian cancer cells, *SPT3 *(serine palmitoyltransferase 3), which has been suggested to make shorter sphingoid base chains [17] and *DES2*, a dihydroceramide desaturase that is bifunctional as a desaturase to produce Cer and as a 4-hydroxylase to produce phytoceramides [18]. We have not noted these backbones in any of the samples analyzed in this study, but this warrants further investigation.

### Variability in gene expression in serous papillary ovarian carcinoma versus normal ovarian stromal tissues

**Figure 2:**
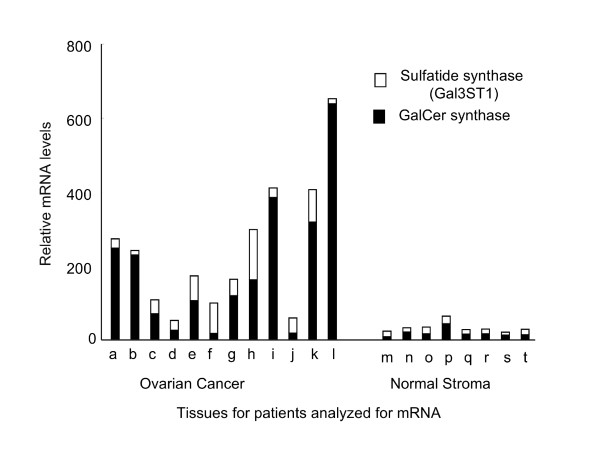
**Individual variation in the microarray data for the synthases for ST and GalCer in ovarian carcinoma versus normal stromal tissue**. Each letter represents the microarray data from an individual subject. The relative mRNA levels for the synthases for ST (*Gal3ST1*) and GalCer were analyzed for normal human ovarian stromal tissues (n = 8) and epithelial ovarian tumor cells (n = 12) using Affymetrix HG U133 Plus 2 Gene Chips.

Since the heat map in Figure 1A depicts the average fold differences, and the data in Figure 1B and 1C show that there is considerable variability in the amounts of ST in samples from different individuals, the individual variability in the microarray results has been shown in Figure 2. *ST synthase *(the white bars) was higher in the cancer samples than the normal tissues in most, but not, all cases (for example, not for patients "b" and "l"); however, *GalCer synthase *(dark bars), was elevated in some of the tumors where *ST synthase *was lower (and vice versa). Since GalCer is the direct precursor of ST, elevation of GalCer synthase activity is also expected to increase the amount of ST. For the tissues analyzed in Figure 1B and 1C, GalCer was on average ~3-fold higher for the cancer tissues (9.4 ± 3.7 pmol/mg protein) than for the normal tissues (2.7 ± 0.6 pmol/mg protein); however, this had only a borderline statistical significance (*P *= 0.13, n = 12 for each group by one-tailed Wilcoxon rank sum test). Therefore, additional studies will be needed to determine if elevation of ST synthase and GalCer synthase are both contributors to the elevation of ST in ovarian cancer.

### Visualization of ST in serous papillary ovarian carcinoma tissues by MALDI TIMS

Ovarian cancer tissue is comprised of a number of cell types, including the ovarian epithelial carcinoma and non-malignant stroma tissue, therefore, MALDI TIMS was used to examine in which areas the increased ST are found. In this method, a MALDI matrix material is deposited in the sample then activated by a laser to generate a plume of ions that ionize some of the compounds in the biological sample, which are mass analyzed. While the ion abundances are not necessarily reflective of the amounts of the analytes because only a fraction of the compounds become ionized in different regions of the tissue, this technique has been useful in examining the localization of a wide range of compounds [19].

**Figure 3:**
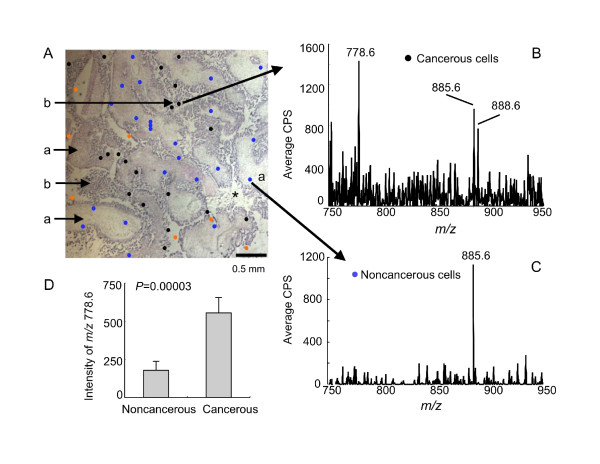
**A representative analysis of a histological thin section from an ovarian carcinoma sample by MALDI tissue imaging mass spectrometry (MALDI TIMS)**. (A) A thin section (10 μm) with H & E staining. Regions distinguished by "a" were histologically identified as non-malignant stroma, "b" as serous papillary epithelial carcinoma, and "*" are regions where there were no cells. The spots represent the sites where the MALDI TIMS spectra were collected (i.e., each spot represents the region targeted by the laser to generate one spectrum) with the regions histologically scored as serous papillary epithelial carcinoma in black; blue spots were from regions scored as stromal cells; the red dots are regions where there were no cells or the histology was undefined. (B and C) Spectra obtained from the shown regions of the adjacent thin section (10 μm). (D) Ion intensities of the *m/z *778.6 (d18:1/C16:0 ST) species from regions scored as normal (n = 23) or ovarian cancer (n = 20) (*P *= 0.00003, by one-tailed Wilcoxon rank sum test).

Figure 3A shows a typical hematoxylin & eosin (H & E) stain of a thin section of ovarian carcinoma tissue showing regions of well-differentiated serous papillary carcinoma with fibrovascular structure lined by micropapillary epithelium with cytologic atypia, loss of nuclear polarity and mitotic figures. Serous papillary carcinoma cells are replacing the ovary and apparently present on the ovarian surface with stromal invasion. A typical fibrovascular stroma is labeled "a" and representative malignant epithelial cells are labeled "b". There are a few spaces with no visible cells, labeled "*". MALDI TIMS analysis of representative malignant epithelial cells gave spectra similar to that in Figure 3B (the spectrum for the specific spot shown), with readily seen ions with *m/z *778.6, 885.6, 888.6 and (in greater amounts in samples than the one shown) 890.6, which correspond to ST with the backbones d18:1/C16:0, an unknown species, d18:0/C24:1, and d18:1/C24:0, respectively. In contrast, spectra from the stromal region (one example shown in 3C) mainly have the ion with *m/z *885.6 and little or no ST. Figure 3D shows a distribution diagram for the intensities of the *m/z *778.6 (d18:1/C16:0 ST) for all of the randomly selected spots shown in Figure 3A with blue for the histologically normal stroma (n = 23) versus black for carcinoma regions (n = 20) (the red spots represent areas where there were no cells or the histology was "undefined"--these regions appear because the spots that were analyzed were picked randomly to avoid bias in the selection of the data for panel D). Using this approach, the mean intensity of *m/z *778.6 in the carcinoma region was 556 ± 99 versus 178 ± 57 in the stroma area (*P *= 0.00003).

### Structural analysis by MALDI tissue-imaging MS/MS

**Figure 4:**
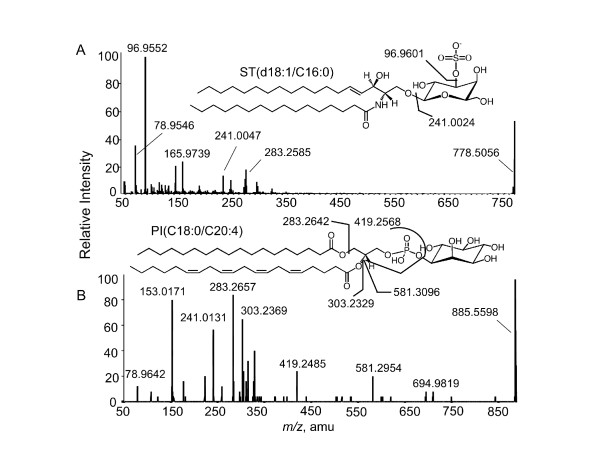
**Identification of ions from MALDI TIMS by precursor ion fragmentation and product ion scan**. A thin section from the ovarian tumor in Fig. 3 was analyzed using a hybrid quadrupole time-of-flight mass spectrometer (ABI Q-STAR) in negative ionization mode with selection of the shown parent ions for fragmentation and analysis of the product ion spectra. (A and B) Product ion spectrum for each compound (*m/z *778.6 ion was identified as a d18:1/C16:0 ceramide monohexosylsulfatide and *m/z *885.6 was most consistent with the shown phosphatidylinositol).

Structural assignments based on MS data alone have the possibility of being in error if the sample happens to contain another compound with the same *m/z *as the compound of interest. The ST subspecies noted in Figure 3 were consistent with the findings from LC ESI-MS/MS analysis of lipid extracts of equivalent tissues, however, one can also confirm the structural assignments by tissue imaging tandem mass spectrometry. For this analysis, the tumor samples were analyzed using ABI Q-STAR and the spectra for the putative ST (d18:1/C16:0, *m/z *778.6) and the unidentified ion with *m/z *885.6 are shown in Figure 4A and 4B, respectively.

The product ion scan spectrum of the ion with *m/z *778.6 in the negative ionization mode gave a highly abundant fragment ion of *m/z *96.9552, corresponding to a sulfate group (HSO_4_). A less abundant fragment ion at *m/z *241.0047 also appears from cleavage to release the sulfated sugar headgroup as shown (Figure 4A). The accurate mass for the presumed precursor ion d18:1/C16:0 ST, [(M-H)^-^, C_40_H_76_N_1_S_1_O_11_], was 778.5056 and was within 15 ppm of the theoretical mass of 778.5144. Similarly, the observed mass for the sulfated carbohydrate (C_6_H_9_SO_8_), 241.0047 and sulfate group, 96.9552, were less than 10 ppm different from the theoretical masses, 241.0024, 96.9601, respectively. Therefore, the structure of *m/z *778.6 is consistent with ST (also called SM4 sulfatide, HSO_3_-3Galβ-1Cer) with a d18:1/C16:0 ceramide backbone. A similar fragmentation has been seen in brain tissue, which is rich in sulfatides [20-22].

The cleavage products from the *m/z *885.6 precursor ion (Figure 4B) included a peak for *m/z *241.0131, but did not show an ion of *m/z *96.9 above background, suggesting that it was not a sulfatide (additionally, the mass difference between what was observed and the theoretical mass of a sulfated sugar is much larger than expected). A much closer fit is obtained when a phosphoinositol head group is considered (C_6_H_10_PO_8_, *m/z *241.0118). A possible structure for this ion has been drawn above the spectrum in Figure 4B (a phosphatidylinositol with C18:0 and C20:4 as the fatty acid) with cleavages that might account for the observed product ions, in agreement with what has been seen by others using MALDI to image other tissues [23].

### Histological localization of ST in serous papillary ovarian carcinoma tissues by MALDI TIMS

**Figure 5:**
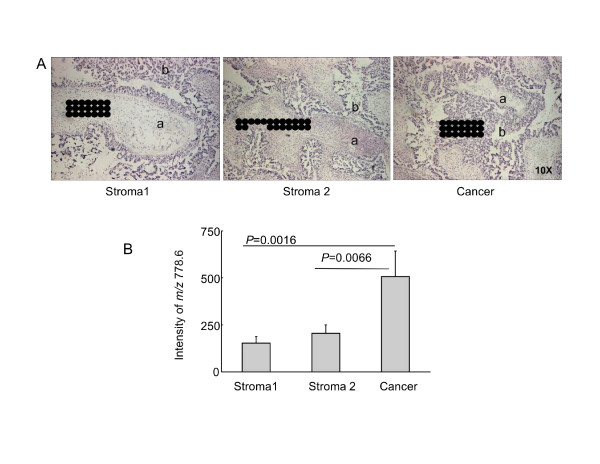
**Comparison of ion intensities for ST in non-malignant stroma or carcinoma as identified by H & E staining**. (A) Regions of H & E staining thin section from Figure 3. The spots represent the sites where the MALDI TIMS spectra were collected in adjacent sections. (B) Distribution of the intensities of the ions (*m/z *778.6 (d18:1/C16:0 ST)) in stromal regions and cancerous regions.

In addition to comparing the ion intensities for randomly selected regions of the tissue slice, we also examined clusters of laser shots through neighboring regions that had stromal and epithelial ovarian carcinoma cells as identified by H & E staining (Figure 5A) (i.e., each dot in this figure represents a region where a MALDI TIMS spectrum was collected), then the intensities of *m/z *778.6 (which corresponds to ST with the backbones d18:1/C16:0) were compared for these specific regions (Figure 5B). Using this approach, the ion intensities of this ST in the stroma (the left and middle panels in Figure 5A) were 153 ± 34 (stroma 1) and 204 ± 44 (stroma 2), which were significantly lower than the intensity in the region identified as epithelial ovarian carcinoma (507 ± 135; *P *= 0.0016 and 0.0066 versus stroma 1 and 2, respectively, n = 21 for each) (right panel of Figure 5A).

**Figure 6:**
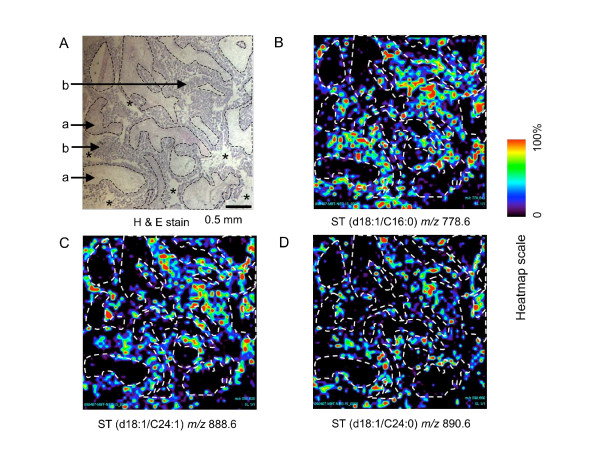
**Visualization of the localization of ST in a thin section of ovarian carcinoma tissue using MALDI TIMS**. (A) H & E stained thin section from Figure 3. Distinctive features in (A) have been manually traced with dashed lines, which have been superimposed on (B-D) to aid in comparison of the distribution of the ions with the H & E stained thin section. (B-D) Pseudo-color ion images where the relatively intensity of the labeled *m/z *778.6 (B), *m/z *888.6 (C), and *m/z *890.6 (D) using the heatmap scale.

The third approach that was used to compare the localization of ST in the entire thin section was to plot the relative ion abundances of the major ST (*m/z *778.6, 888.6 and 890.6 correspondingly to d18:1/C16:0-, d18:1/C24:1-, and d18:1/C24:0-ST, respectively) collected at 60 μm intervals throughout the entire x, y field, as shown in Figure 6. As noted above for the H & E stained image (Figure 6A), there are regions with typical fibrovascular stroma ("a") and malignant epithelial cells ("b") as well as a few spaces with no visible cells ("*"). For ease of comparison, dashed lines have been traced around the stromal regions and these have been placed over the images from the MALDI TIMS analyses in Figure 6B-D. All three ST are seen in most of the tissue regions identified as ovarian epithelial carcinoma, in strong contrast with the stromal regions, which were essentially free of ions with *m/z *778.6, 888.6 or 890.6 (Figure 6B-D).

### Comparison of gene expression and ST visualization for serous papillary ovarian carcinoma versus normal ovarian surface epithelial cells

**Figure 7:**
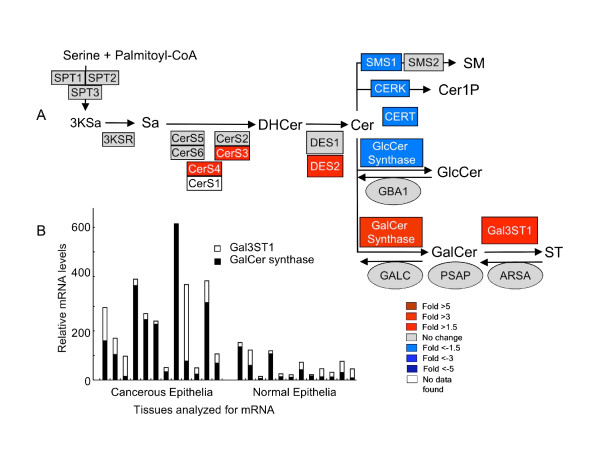
**Difference in the level of expression of genes for sphingolipid biosynthesis through ST of human ovarian normal surface epithelial cells and epithelial tumor cells**. The relative mRNA levels were analyzed for normal human ovarian surface epithelial cells (n = 12) and epithelial ovarian tumor cells (n = 12) using Affymetrix HG U133 Plus 2 Gene Chips. The fold differences of mRNA level for the shown steps of the *de novo *sphingolipid biosynthesis pathway were imported into a KEGG style pathway heatmap. (A) Fold difference in gene expression for ovarian epithelial carcinoma/normal epithelial cells are represented by the color scale (red = higher; blue = lower). (B) Relative mRNA levels for *Gal3ST1 *and *GalCer synthase *in each of the normal ovarian surface epithelial cells (n = 12) and ovarian epithelial carcinoma (n = 12).

Since the comparison in Figure 1 was between epithelial ovarian cancer cells versus stromal cells, it is possible that the differences might reflect the nature of ovarian epithelial cells and stroma, rather than the ovarian carcinoma *per se*. Figure 7 shows a comparison of the gene expression profiles for healthy ovarian epithelial cells harvested from normal ovaries at the time of surgery using a pap brush and ovarian carcinoma epithelial cells collected by laser capture microdissection and, as was seen in comparing the cancerous versus normal stormal tissues, *Gal3ST1*and *GalCer synthase *are higher in ovarian epithelial carcinoma cells (by 5- and 2.3-fold, respectively) but *ARSA*, *GALC and PSAP *were not noticeably different (Figure 7A). The *Gal3ST1 *and *GalCer synthase *gene expression differences for the individual subjects are also shown in Figure 7B and, as was seen in the comparison with stromal tissues (Figure 2), there was considerable variation among individuals with some displaying elevations in mainly *Gal3ST1*, others in *GalCer synthase*, and some with both. Overall, however, these gene expression data predict that ST and GalCer are higher in most ovarian epithelial carcinoma cells versus normal surface epithelial cells.

**Figure 8:**
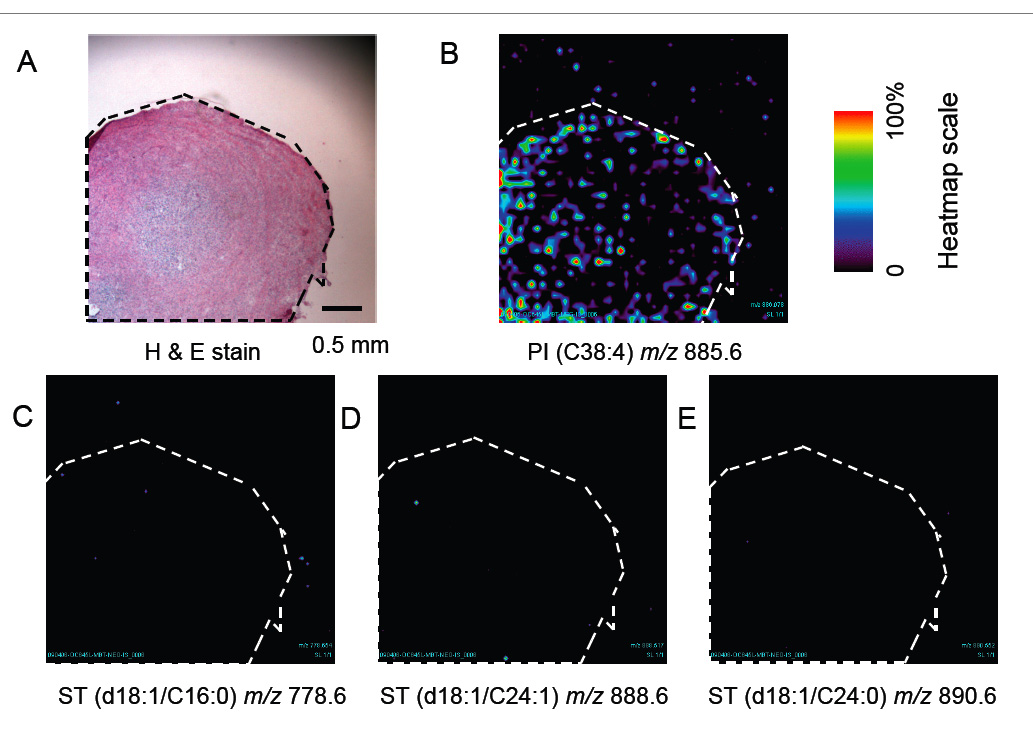
**Visualization of phosphatidylinositol and ST in a thin section of normal ovarian tissue using MALDI TIMS**. Adjacent thin sections of normal ovarian tissue was prepared as described in the text for H & E staining (A) and pseudo-color ion images for phosphatidylinositol (B) as a positive control (*m/z *885.6 at the instrument calibration used for this image) and ST (at *m/z *778.6, C; *m/z *888.6, D; and *m/z *890.6, E) using the shown heat map scale. The ovarian tissue in (A) has been manually traced with dashed lines, which have been superimposed on panels B-E to aid in comparison.

MALDI TIMS was also used to analyze a thin section from normal ovarian tissue. The image is shown in Figure 8, illustrating that the major ST (*m/z *778.6, 888.6 and 890.6) were not detectable, whereas PI (*m/z *885.6) was detectable and served as a positive control (Figure 8B).

## Discussion

By combining complementary profiling technologies, metabolite analysis by mass spectrometry with analysis of gene expression by laser capture microdissection, this study achieved its original goal of rigorously establishing that ST are elevated in ovarian cancer, as had been suggested by an earlier investigation [9]. LC ESI-MS/MS provided quantitative and structure-specific information about the ST as well as its precursor GalCer in the tumors, and MALDI TIMS localized the ST to regions identified as ovarian epithelial carcinoma versus surrounding stromal and normal ovarian tissue. The gene expression analysis supported this conclusion by finding higher amounts of mRNA's for both of the key enzymes of ST biosynthesis, *GalCer synthase *and *Gal3ST1*, in the epithelial ovarian carcinoma cells versus normal ovarian stromal tissue and normal ovarian surface epithelial cells; whereas, the mRNA for three enzymes of ST catabolism, *GALC *and *ARSA*, and *PSAP *were not different. In addition, the carcinoma cells had greater expression of the mRNA's for two Cer synthases (*CerS3 *and *4*) that produce very-long-chain-Cer, which were prevalent in the ST of most of the ovarian cancer samples. Thus, the elevation of very-long-chain-ST in epithelial ovarian carcinoma cells is likely a consequence of these gene expression changes.

It is interesting that some subjects have elevations in mRNA for *GalCer synthase *but not *Gal3ST1 *(Figure 2). This suggests that GalCer might accumulate when there is insufficient *Gal3ST1 *to convert it into ST and possibly account for the finding in another type of ovarian cancer, mucinous cystadenocarcinoma, that both GalCer, and to a lesser extent ST, are elevated [24].

The consequence(s) of the elevation in ST in ovarian cancer are not known, but by analogy might affect the metastatic potential of tumors because ST and other sulfated glycolipids have been reported to participate in the metastasis of colorectal adenocarcinoma [5] and KHT fibrosarcoma cells [25]. ST are thought to facilitate metastasis as ligands for selectins [26], but they also interact with other extracellular proteins such as laminin and thrombospondin [27]. Other microarray data from this study (not shown) also indicated that P-selectin mRNA expression is 1.5-fold higher in ovarian carcinoma epithelial cells versus normal ovarian surface epithelial cell, and L-selectin is 2.5-fold higher in cancer tissue than normal stromal tissue; therefore, both the ST ligands and the binding partners for them appear to be elevated. ST additionally affect the immune system, altering the phenotype of macrophages [28], which have been associated with ovarian cancer invasion and metastasis [29]. It should also be borne in mind that some of the cancer-relevant targets of ST might be intracellular because ST bind to the N-terminal domain of sphingosine kinase 2 [30], an enzyme that produces sphingosine 1-phosphate and plays many important roles in cancer [31], including regulation of histone acetylation [32]. The presence of ST in ovarian epithelial carcinoma cells might also have the practical advantage of providing a biotargeting ligand for anti-tumor nanoparticles [33].

ST might have utility as histologic biomarkers, particularly if their elevation reflects an important tumor phenotype, such as metastatic potential. As mass spectrometry becomes more widely applied to biopsy samples, as has been proposed [34], ST might be useful ions to monitor since they have been suggested to reflect early stages of ovarian cancer [9], and to be elevated in a wide range of other cancers, including colorectal [5], hepatocellular [6], renal [7], brain [35] and small-cell lung cancers [8]. It is appealing to imagine that there might be molecular markers, such as ST, that are detectable by MALDI TIMS analysis of biopsy samples and allow identification of early tumors that would otherwise be incorrectly scored as "normal" by histologic staining alone. The possibility that ST might be useful as serum biomarkers also warrants further study.

## Materials and methods

### Tissues

For microarray analysis of gene expression in the ovarian cancer epithelial cells and normal ovarian surface epithelial cells, serous papillary ovarian cancer samples from 12 patients with a mean age of 59 years (range 48-71, stages Ic-IV) were collected during surgery, sealed in cryotubes and frozen in liquid nitrogen in less than one minute; for the normal ovarian surface epithelial cells, surface epithelial cells were collected from 12 controls (mean age 53 years, range 41-78, who were in the clinic for other conditions) using a pap brush and stored in RNAlater solution (Ambion, Austin, TX) at -20°C. For microarray analysis of gene expression in the ovarian cancer epithelial cells and normal ovarian stromal cells, the same data set for serous papillary ovarian cancer samples from 12 patients (see above) was used; for the normal ovarian stromal cells, ovarian tissues were collected during surgery from eight of the controls described above (mean age 52 years, range 41-63), sealed in cryotubes and frozen in liquid nitrogen in less than one minute.

For mass spectrometric analyses (LC ESI-MS/MS and MALDI TIMS), 12 serous papillary ovarian cancer tissues from other women (mean age 59 years, range 46-71, categorized as stages IIa-IV ovarian cancer) and 12 histological normal ovarian tissues (from women with a mean age of 54 years, range 36-84) were collected during surgery, sealed in cryotubes and frozen in liquid nitrogen in less than one minute. Controls were defined as patients at Northside Hospital (Atlanta, GA) with ovarian histology considered within normal limits, WNL, and women with non-cancerous ovarian conditions. All of the samples were collected at Northside Hospital and later transported to Georgia Institute of Technology on dry ice, and stored at -80°C for future use. All of the work in this project followed Georgia Institute of Technology and Northside Hospital IRB approved protocols.

### Sphingolipid analysis by LC ESI-MS/MS

The tissues (typically in the range of 0.7 to 1.6 mg) were prepared as 10% homogenates (*w/v*, in distilled, deionized water) and extracted for analysis of GalCer and ST by mass spectrometry as previously described [12,36].

After addition of the solvents to the tissue homogenate, an internal standard cocktail consisting of 25 pmol of C12-ST and C12-glucosylceramide (Avanti Polar Lipids, Alabaster, AL) was added. The LC ESI-MS/MS analysis was conducted using a Perkin Elmer Series 200 autoinjector, and a Shimadzu LC-10 AD VP binary pump system coupled to a 4000 quadrupole linear-ion trap (QTrap) (Applied Biosystems, Foster City, CA).

For GalCer analysis, the lower phase organic extract was resuspended in 300 μl of mobile phase, representing about 1 mg of original tissue, then 30 μl were analyzed by LC ESI-MS/MS using a Supelco 2.1 mm × 25 cm SUPELCOSIL LC-Si column (Sigma, St. Louis, MO) with isocratic elution at 1.5 ml/min using a mobile phase consisting of CH_3 _CN:CH_3 _OH:CH_3 _COOH (97:2:1, *v:v:v*) with 5 mM ammonium acetate. For every run, the column was equilibrated for 1.5 min prior to injection, the sample was injected and eluted for 8 min (with GlcCer, and GalCer eluting at ~ 3 min and 3.5 min, respectively, with baseline resolution), followed by re-equilibration of the column for the next run. Resolution of these isomers was confirmed during the analysis by interspersing vials with internal standards (C12GalCer and C12GlcCer) throughout the runs.

For ST analysis, the extract was resuspended in 300 μl of LC solvent (CH_3_OH:H_2_O, 95:5, *v:v*, with 5 mM ammonium acetate and 0.01% NH_4_OH), representing about 1 mg of original tissue, then 50 μl was analyzed by reverse-phase LC ESI-MS/MS using a 2.1 × 20 mm Ace C18 column (MAC-MOD Analytical, Chadds Ford, PA) eluted at flow rate of 0.5 ml/min. The column was first equilibrated with a 10:90 (*v:v*) mixture of mobile phase solvent A (CH_3_OH:H_2_O, 50:50, *v:v*, with 5 mM ammonium acetate and 0.01% NH_4_OH) and solvent B (CH_3_OH with 5 mM ammonium acetate and 0.01% NH_4_OH) for 2 min, then sample was injected and eluted with this mixture for 1 min, followed by a gradient to 100% solvent B over 3 min, then sustained at 100% solvent B for 5 min, during which the different subspecies of ST elute (between ~ 4 and 7 min); finally, the solvent was restored to the original A:B mixture (10:90, *v:v*) by a 1 min gradient, and equilibrated for 2 min before the next run.

The declustering potential (DP) and entrance potential (EP) for the API 4000 QTrap were adjusted to achieve the optimal ionization conditions. After the Q1 settings were determined, product ion spectra were collected across a range of collision energies (CE), structurally specific product ions were identified, and collision energies and collision cell exit potentials (CXP) were manipulated to produce optimal signal. For GalCer, DP was 35.0 V, EP was 10.0 V, CXP was 15 V, CE was from 50 to 70 V. For ST, DP was -220.0 V, EP was -10.0 V, CXP was -14.0 V, CE was from -55 to -130 V.

The lipid extracts were initially examined for GalCer species by a precursor ion scan of *m/z *264.4 in positive ionization mode, and for ST by a precursor ion scan for precursors for *m/z *96.9, which is specific for the HSO_4 _moiety in negative ionization mode. The resulting precursor/product pairs were used for quantitative analysis by multiple reaction monitoring (MRM) [12]. This preliminary analysis found that the only detectable subspecies were the sulfated monohexosylceramide HSO_3_-3Galβ-1Cer and GalCer with the Cer backbones listed below. Using this information, Q1 and Q3 were set to cycle through these precursor and product ions pairs with a dwell time of 25 ms for each GalCer transition and 20 ms for each ST transition and an interchannel delay of 5 ms between transitions. For GalCer, the transitions occur at *m/z *with the nature of the lipid backbone of the sphingolipid in parentheses (sphingoid base carbon number:number of double bonds/fatty acid carbon number:number of double bonds): 700.7/264.4 (d18:1/16:0), 728.7/264.4 (d18:1/18:0), 756.7/264.4 (d18:1/20:0), 784.8/264.4 (d18:1/22:0), 810.9/264.4 (d18:1/24:1), 812.9/264.4 (d18:1/24:0), 838.9/264.4 (d18:1/26:1), and 840.9/264.4 (d18:1/26:0); for ST, the transitions occur at the following *m/z *778.6/96.9 (d18:1/16:0), 806.6/96.9 (d18:1/18:0), 834.5/96.9 (d18:1/20:0), 862.6/96.9 (d18:1/22:0), 888.6/96.9 (d18:1/24:1), 890.6/96.9 (d18:1/24:0), 916.6/96.9 (d18:1/26:1), and 918.6/96.9 (d18:1/26:0). The quantity of each subspecies was determined by comparison of the areas for each MRM transition with the areas of the spiked internal standards as previously described [12,36]. Tissue extracts were normalized by the protein amount in the tissue homogenate using the BCA method (Thermo Sci, Rockford, IL).

### MALDI tissue-imaging mass spectrometry

The tissue distribution of ST was determined essentially as described by others [37,38], with recent modifications [13,39]. Starting with ovarian tissues that had been frozen in liquid nitrogen and stored at -80°C, the frozen tissue was put into a sealed dry-ice box for 60 min, mounted in a LEICA CM3050 S cryostat (LEICA, Germany) at -20°C for ~30 min, then sectioned into 10 μm thick slices (avoiding folding) and thaw-mounted onto MALDI plates (Applied Biosystems, Foster City, CA) at -20°C, then stored at -80°C until analysis (within a month). Neighboring 10-μm slices were thaw-mounted into glass slides for H & E staining. To analyze the tissue slices by TIMS, the tissue slices on the MALDI plate were slowly brought to room temperature in a desiccator before a matrix solution (2-mercaptobenzothiazole, from Sigma, at 5 mg/mL in methanol) [39] was sprayed onto the sample using an oscillating capillary nebulizer sprayer (OCN) with a syringe pump (Harvard, Canada) as described previously [13]. The typical time required for matrix coating of a 4 cm^2 ^sample was ~45 min.

Mass spectra for imaging were acquired using a Voyager DE STR MALDI-TOF mass spectrometer (Applied Biosystems, Foster City, CA) with a 337 nm N_2 _laser (3 Hz) under delayed extraction conditions in reflector mode. The accelerating voltage, grid voltage, and delay time were 20 kV, 72% and 220 ns, respectively. MALDI TIMS data sets were acquired using modified MMSIT (MALDI MS Image Tool) (Applied Biosystems) (without 32k data limitation) over the tissue section. Twelve laser shots were summed for each sample spot and the step size of sample stage was 60 μm. Ion images were reconstituted using BioMap software package (Novartis Pharma AG, Basel, Sweden).

For comparison of the ion intensities of ST in different regions of the tissue slice, sample spots (60 μm) were chosen as described in the text. The ion intensity for the *m/z *of interest is analyzed by one-tailed Wilcoxon rank sum test with *P *< 0.05 considered to be significantly different.

The major ions of interest (i.e., putative ST) underwent further structural analysis using a hybrid quadrupole time-of-flight mass spectrometer (Q-STAR, Applied Biosystems Foster City, CA) equipped with an O-MALDI source using a 337 nm N_2 _Laser (30 Hz). The laser energy and instrument parameters were optimized for MS/MS analysis of the fragmentation of the precursor ion to informative product ions. Briefly, the DP was 0.0, Focusing Potential (FP) was -80.0, DP2 was -15.0, CE was varied from -20 to -80 V, Collision Gas (CAD) was 6, Ion Energy 1(IE1) was -0.9, DC Quad Lens Horizontal Focus (GR) was -5.0, and laser relative intensity was 22%.

### RNA isolation, amplification and microarray analysis

For analysis of gene expression in the ovarian cancer epithelial cells and normal ovarian stromal tissue, the frozen tissues were embedded in cryomatrix (Shandon, Thermo Fisher scientific, Waltham, MA) and sectioned into 7-μm thick frozen slices using a cryostat and were subsequently attached to uncharged microscope slides. Immediately following dehydration and staining (HistoGene LCM Frozen Section Staining Kit, Arcturus, Molecular Devices, Sunnyvale, CA), slides were placed in an AutoPix™ LCM instrument (Arcturus) for laser capture microdissection of cells to CapSure Macro LCM Caps (Arcturus). Approximately 30,000 epithelial cells were collected from each of the twelve cancer samples, and normal stromal tissue were collected from each of the 8 normal ovarian tissues, then RNA was extracted in 25 μL of extraction buffer using the PicoPure RNA Isolation Kit (Arcturus). RNA was isolated from the normal surface ovarian epithelial cells stored in RNAlater solution using the RNAlater kit (Ambion, Foster City, CA). Biotin labeled mRNA was prepared from the previously isolated mRNA from tumor and normal cells using the RiboAmp OA or HS kit (Arcturus) in conjunction with the IVT Labeling Kit (Affymetrix, Santa Clara, CA) for hybridization to Human Genome U133 Plus 2.0 Array GeneChips (Affymetrix) for 3' expression analysis. The arrays were processed following the GeneChip → Expression Technical Manual to generate feature level expression results (.CEL files). The .CEL files were processed to generate a probe set summarization file by the Affymetrix Expression Console (EC) Software Version 1.1 software, which uses the default MAS5 3' expression workflow which scaled all probe sets to a target intensity (TGT) of 500. AFFX-BioB, AFFX-BioC, AFFX-BioDn, and AFFX-CreX were used as the spiked internal standard controls.

### Analysis and visualization of the gene expression data using a GenMapp pathway diagram for sphingolipids

The expression levels for the genes of interest were extracted from the probe set summarization file. The average fold difference for each gene was calculated for the normal ovarian surface epithelia (n = 12) or normal ovarian stroma (n = 8) versus the serous papillary ovarian cancer epithelia (n = 12) using a per script (ActivePerl v5.8, ActiveState, Vancouver, BC). The results were visualized using GenMapp v2.1 [40], a program for the creation and visualization of gene expression data related to a specific biochemical or signaling pathway in a KEGG pathway pattern [14], as recently modified for sphingolipids [15]. The fold-differences were visualized by the color criteria (heatmap) shown in the figure.

### Statistical analysis

The statistical analysis was performed using software R 2.8.1 (R-project.org). Data were analyzed using one-tailed Wilcoxon rank sum test. The results were considered statistically significant if *P *< 0.05.

## Abbreviations

ARSA: arylsulfatase; CerS: ceramide synthase; GALC: galactosylceramidase; GalCer: galactosylceramide; Gal3ST1: 3'-phosphoadenosine-5'-phosphosulfate:GalCer sulfotransferase; LC ESI-MS/MS: liquid chromatography electrospray ionization-tandem mass spectrometry; MALDI TIMS: matrix-assisted laser desorption/ionization tissue-imaging mass spectrometry; PSAP: prosaposin; ST: sulfatide.

## Competing interests

The authors declare that they have no competing interests.

## Authors' contributions

YL carried out sphingolipid analysis by LC ESI-MS/MS, portions of MALDI tissue-imaging mass spectrometry and wrote the initial drafts of the manuscript. YC carried out portions of MALDI tissue-imaging mass spectrometry. AM prepared the pathway diagram to visualize the sphingolipid related gene expression. RS and EW were involved in some of sphingolipid analysis by LC ESI-MS/MS. NJB analyzed the microarray data. LVM and LDW carried out the RNA isolation, amplification and hybridization to Genechips. JFM was the principal investigator for the collection of the human samples and microarray analysis, and contributed to the writing of the manuscript. MCS contributed to the experimental design and writing of the manuscript. AHM contributed to the conception and design of the entire study and the final editing of the manuscript. All authors read and approved the final manuscript.
